# Microbiota-Derived Tryptophan Metabolite Indole-3-Propionic Acid-Emerging Role in Neuroprotection

**DOI:** 10.3390/molecules30173628

**Published:** 2025-09-05

**Authors:** Maja Owe-Larsson, Dominik Drobek, Paulina Iwaniak, Renata Kloc, Ewa M. Urbanska, Mirosława Chwil

**Affiliations:** 1 Department of Histology and Embryology, Center of Biostructure Research, Medical University of Warsaw, Chałubińskiego 5, 02-004 Warsaw, Poland; maja.owe-larsson@wum.edu.pl; 2 Chair and Department of Experimental and Clinical Pharmacology, Medical University of Lublin, Jaczewskiego 8b, 20-090 Lublin, Poland; ddrobek0@icloud.com (D.D.); paulina.iwaniak@umlub.edu.pl (P.I.); renata.kloc@umlub.edu.pl (R.K.); 3 Department of Botany and Plant Physiology, University of Life Sciences in Lublin, Akademicka 15, 20-950 Lublin, Poland; miroslawa.chwil@up.lublin.pl

**Keywords:** indole-3-propionic acid, neuronal loss, microbiota, tryptophan, kynurenic acid, Alzheimer’s disease, Parkinson’s disease, stroke, inflammation, aryl hydrocarbon receptor, pregnane X receptor

## Abstract

In recent years, gut–brain axis signaling has been recognized as an essential factor modifying behavior, mood, cognition, and cellular viability under physiological and pathological conditions. Consequently, the intestinal microbiome has become a potential therapeutic target in neurological and psychiatric disorders. The microbiota-derived metabolite of tryptophan (Trp), indole-3-propionic acid (IPA), was discovered to target a number of molecular processes and to impact brain function. In this review, we outline the key mechanisms by which IPA may affect neuronal activity and survival and provide an update on the evidence supporting the neuroprotective action of the compound in various experimental paradigms. Accumulating data indicates that IPA is a free radical scavenger, a ligand of aryl hydrocarbon receptors (AhR) and pregnane X receptors (PXR), and an anti-inflammatory molecule. IPA decreases the synthesis of the proinflammatory nuclear factor kappa-light-chain-enhancer of activated B cells (NF-κB), tumor necrosis factor-α (TNF-α), and other cytokines, reduces the generation of the NLR family pyrin domain containing 3 (NLRP3) inflammasome, and enhances the synthesis of neurotrophic factors. Furthermore, produced in the gut, or administered orally, IPA boosts the central levels of kynurenic acid (KYNA), a neuroprotective metabolite of Trp. IPA reduces the release of proinflammatory molecules in the gut, breaking the gut–inflammation–brain vicious cycle, which otherwise leads to neuronal loss. Moreover, as a molecule that easily enters central compartment, IPA may directly impact brain function and cellular survival. Overall, the gathered data confirms neuroprotective features of IPA, and supports its potential use in high-risk populations, in order to delay the onset and ameliorate the course of neurodegenerative disorders and cognitive impairment. Clinical trials evaluating IPA as a promising therapeutic add-on, able to slow down the progress of neurodegenerative disorders such as Alzheimer’s or Parkinson’s disease and to limit the morphological and behavioral consequences of ischemic stroke, are urgently needed.

## 1. Introduction

**Figure 1:**
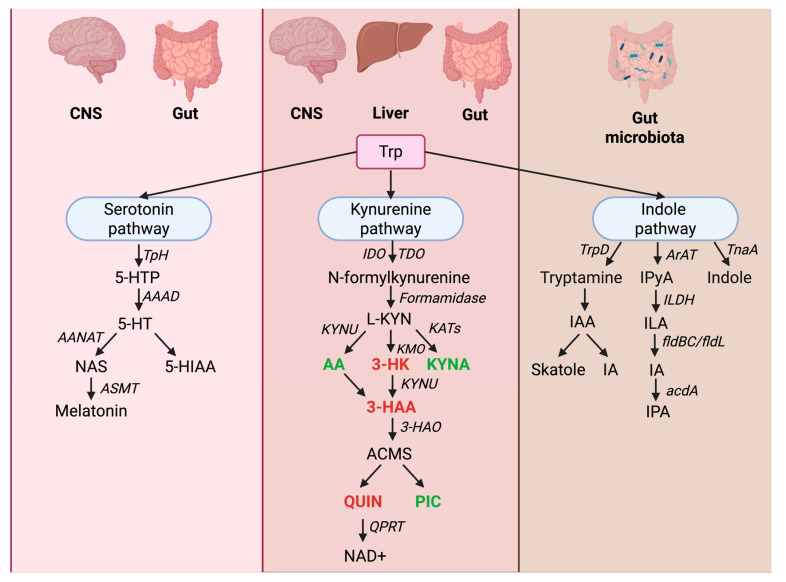
Tryptophan metabolic pathways. Abbreviations: 3-HAA, 3-hydroxyanthranilic acid; 3-HAO, 3-hydroxyanthranilate 3,4-dioxygenase; 3-HK, 3-hydroxykynurenine; 5-HIAA, 5-hydroxyindoleacetic acid; 5-HT, 5-hydroxytryptamine; 5-HTP, 5-hydroxytryptophan; AA, anthranilic acid; AAAD, aromatic L-amino acid decarboxylase; AANAT, arylalkylamine N-acetyltransferase; ACMS, aminocarboxymuconate semialdehyde; acdA, acyl-CoA dehydrogenase A; ArAT, aromatic amino acid transaminase; ASMT, acetylserotonin O-methyltransferase; CNS, central nervous system; fldBC, phenyllactate dehydratase BC; fldL, phenyllactate dehydratase L; IA, indoleacrylic acid; IAA, indole-3-acetic acid; IDO, indoleamine 2,3-dioxygenase; ILDH, indolelactate dehydrogenase; ILA, indolelactic acid; IPyA, indole-3-pyruvic acid; IPA, indole-3-propionic acid; KATs, kynurenine aminotransferases; KMO, kynurenine 3-monooxygenase; KYNA, kynurenic acid; KYNU, kynureninase; L-KYN, L-kynurenine; NAD^+^, nicotinamide adenine dinucleotide (oxidized form); NAS, N-acetylserotonin; PIC, picolinic acid; QPRT, quinolinate phosphoribosyltransferase; QUIN, quinolinic acid; TDO, tryptophan 2,3-dioxygenase; TnaA, tryptophanase; TpH, tryptophan hydroxylase; Trp, tryptophan; TrpD, anthranilate phosphoribosyltransferase (trpD gene product). Created in BioRender. Drobek, D. (2025) https://BioRender.com/0c885zc (accessed on 2 September 2025).

The importance of the gut–brain axis in the functioning of the central nervous system is well-recognized and broadly accepted. Intriguing discoveries of the last decades implicate the intestinal microbiome as a potential therapeutic target in neurological and psychiatric disorders [1,2,3,4]. In the gut, tryptophan (Trp), an essential amino acid obtained from dietary sources, undergoes metabolic conversion by host and microbiota cells. Intestinal metabolism of Trp involves three major pathways (Figure 1). Trp is a substrate for the production of (A) serotonin by enterochromaffin cells and microbiota, (B) so-called kynurenines along the kynurenine pathway (KP) by immunocompetent and epithelial cells, and (C) various bioactive compounds, mostly indoles, by microbiota [5,6]. It is estimated that 4–6% of dietary Trp is metabolized along the indole pyruvate pathway [6]. The most abundant metabolite is indole, followed by indole-3-acetic acid (IAA) and indole-3-propionic acid (IPA) [7,8,9]. Emerging data demonstrate that indoles exert a profound impact on metabolic, immune, cardiovascular, and brain function [10]. Microbiota-derived IPA possesses strong antioxidant and neuroprotective properties, and may ameliorate metabolic, inflammatory, and neurodegenerative changes in various experimental paradigms [1,2,3,4].

This narrative review aims to present the current state of knowledge on IPA targets and discuss their potential benefits and drawbacks in the treatment of neurodegeneration.

## 2. IPA Synthesis and Tissue Levels

IPA is primarily of bacterial origin. It is synthesized by species populating the soil and by intestinal microbiota residing in the human gut. IPA-producing bacteria originate from the *Clostridiaceae* and *Peptostreptococcaceae* families [10]. Microbial phyla firmicutes (*Roseburia*, *Dorea*, *Butyricicoccus*, *Ruminococcus*, *Eubacterium*, *Faecalibacterium*, *Gemmiger*, *Intestinimonas*, *Sporobacter*, *Oscillibacter*, *Clostridium_XlVb*, and *Coprococcus*), actinobacteria (*Adlercreutzia*, *Collinsella*), bacteroidetes (*Prevotella*, *Alistipes*, *Odoribacter*), and verrucomicrobia (*Akkermansia*) were positively linked with serum IPA levels in a cohort of 318 adults [11]. The mechanisms underlying the metabolism of Trp to IPA are analogous to the processes by which *C. sporogenes* converts phenylalanine into phenylpropionic acid. The specific fldC gene associated with IPA production was identified, and the knockout of the fldC gene completely abolished the generation of IPA [12].

IPA synthesis depends predominantly on the activity of Trp aminotransferase (TAA/ArAT, aromatic amino acid aminotransferase) [13]. ArAT converts Trp to indole-3-pyruvic acid (IPyA), which is later reduced to indolelactic acid (ILA) by indolelactate dehydrogenase (ILDH). ILA is dehydrated to indoleacrylic acid (IA) by phenyllactate dehydratase BC (fldBC) and its activator, acyl-CoA ligase. The final product of Trp reductive metabolism, IPA, is produced from IA by acyl-CoA dehydrogenase (acdA) [9,12] (Figure 1).

The circulating levels of IPA depend on the diversity of intestinal microbiota, the level of Trp supply, the type of ingested food, and the relative activity of other metabolic pathways that convert Trp. IPA can also be found in some food products, such as various plant species of the family Brassicaceae or female asparagus plants (*Asparagus officinalis* L.) [12,14,15].

The impact of nutritional factors on the intestinal milieu, bacterial diversity, and, subsequently, IPA synthesis was shown by several studies. In a large cohort of individuals (N = 1018), the microbiome alpha-diversity was linked with serum IPA levels [16]. In line with this observation, in women with low IPA levels and polycystic ovary syndrome, the alpha-diversity of microbiota and dietary intake of whole-grain cereals were lower [17]. The impact of diet was demonstrated in healthy volunteers fed a high-fat, low-fiber diet for a short period. This resulted in an altered ratio of intestinal microbiota species and a subsequent decrease in IPA synthesis [18]. In another study, fiber and carbohydrate intake were correlated with circulating levels of IPA in healthy controls and individuals predisposed to diabetes [19]. Consequently, prolonged administration of probiotic strains of *Bifidobacterium* in a randomized, double-blind clinical trial (N = 31) resulted in an almost two-fold increase in serum IPA level. Furthermore, a Trp-rich diet potently increases peripheral levels of IPA [20,21].

IPA, generated by microbiota, enters epithelial cells, where it can reach its first targets in the gut–brain axis, such as the aryl hydrocarbon receptor (AhR) and pregnane X receptor (PXR), which are primarily involved in immune response modulation. Upon absorption from the GI, IPA is distributed systemically, and, in the process of simple diffusion, easily crosses the blood–brain barrier. It was discovered that brain IPA is not produced in situ, but derives from systemic circulation, and microbial metabolism of Trp to indole derivatives is indispensable in this process [14,22,23].

The levels of IPA in body fluids vary between species. In rats, IPA concentration reached approximately 5 μM in the blood [24]. In humans, serum IPA levels are approximately 100–200 ng/mL, i.e., a 0.5–1 μM concentration [10,23]. Clinical data from various cohorts revealed control serum levels of IPA from 107 to 190 ng/mL (0.5–1.3 μM), and its decrease in Huntington’s disease (HD) or Parkinson’s disease (PD) by 40–50% [25,26,27].

Studies on the cerebrospinal fluid (CSF) level of IPA are scarce. Human CSF IPA is much lower than in serum and was estimated at 0.63 ± 0.09 ng/mL, i.e., approximately 3 nM [14]. In rats, IPA levels reach 20 ng/mL (approximately 0.1 μM) [28]. In the brain, IPA levels are slightly lower; however, peripheral administration of this compound evokes a 2–3-fold rise, as shown in experimental animals [14,28,29,30].

Consequently, diet composition should be taken into consideration when planning studies and analyzing the impact of gut-derived IPA on brain function.

## 3. Targets of IPA

**Figure 2:**
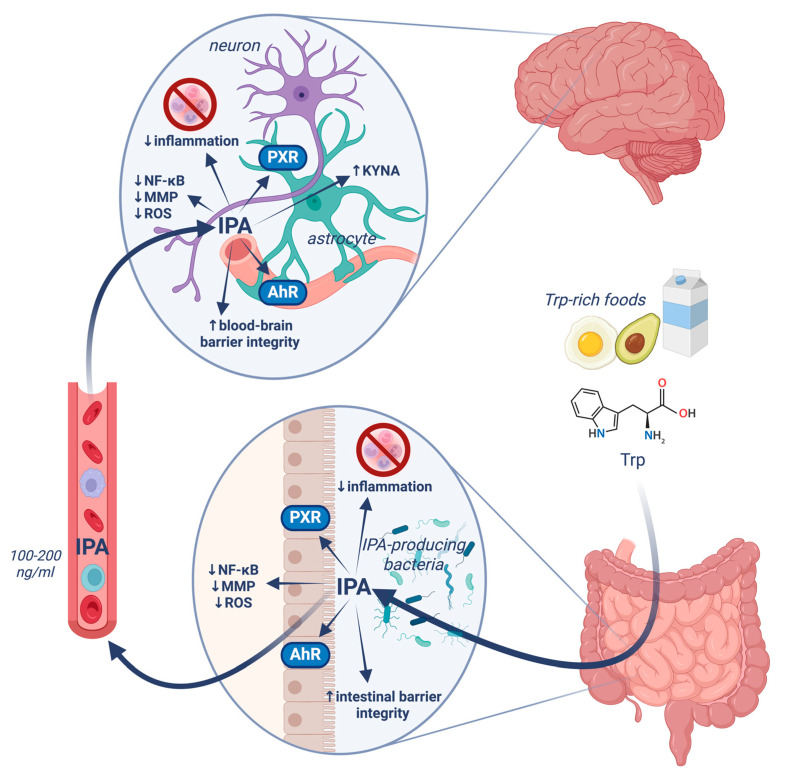
IPA targets. Abbreviations: AhR, aryl hydrocarbon receptor; IPA, indole-3-propionic acid; MMP, matrix metalloproteinase; NF-κB, nuclear factor kappa-light-chain-enhancer of activated B cells; PXR, pregnane X receptor; ROS, reactive oxygen species; Trp, tryptophan. Created in BioRender. Owe-larsson, M. (2025) https://BioRender.com/7axblh2 (accessed on 2 September 2025).

IPA modulates human metabolism, immune response, and cardiovascular and brain function through various molecular targets. Accumulating data have shown that IPA acts as a free radical scavenger, a ligand of aryl hydrocarbon receptors (AhR) and pregnane X receptors (PXR). It improves the integrity of the intestinal barrier and alleviates inflammation [10,31,32]. A correlation between serum IPA and brain-derived neurotrophic factor (BDNF) was demonstrated [33]. Furthermore, orally administered IPA may increase the level of kynurenic acid (KYNA), a neuroprotective metabolite of Trp [34] (Figure 2, Table 1, Table 2).

### 3.1. Aryl Hydrocarbon Receptor

Aryl hydrocarbon receptor (AhR) is an 848-amino-acid transcription factor encoded by the *AHR* gene on chromosome 7p21.1 in humans [61,62,63,64]. Inactive cytoplasmic AhR forms a complex with cochaperone p23, a heat-shock protein, an AhR-interacting protein, and an SRC protein kinase. Upon binding an AhR ligand, the complex undergoes conformational changes, translocates to the nucleus, and there controls the transcription of a variety of genes [62]. The major role of AhR was initially ascribed to xenobiotic detoxification and protection against environmental toxins. Currently, it is broadly accepted that the role of AhR in human physiology is quite complex. The net outcome of its activation depends on the cell type, ontogenetic period of life, and the presence or absence of other signaling molecules/pathogenetic factors [65,66].

Apart from regulating transcription, AhR may target the nuclear factor kappa-light-chain-enhancer of activated B cells (NF-κB), estrogen, or retinoic acid receptors [63,67,68]. AhR may also act as a ubiquitin ligase, which impacts the processes of ubiquitination and proteasomal degradation, and contributes to the regulation of cell division [69]. AhR changes histone acetylation and methylation and influences the long non-coding RNA and microRNAs [63,68]. Furthermore, activation of AhR may impact the metabolism of Trp along the kynurenine pathway. The protein kinase SCR, released upon ligand binding to AhR, phosphorylates the indoleamine 2,3-dioxygenase 1 (IDO1), and, thus, affects the fate of Trp metabolism [70].

In peripheral organs, AhR is expressed in vascular cells and impacts the endothelial barriers, including the gut–brain axis [67,68]. It is considered essential for the balance between the development of antigen tolerance, on one side, and the immune defense against pathogenic microbial species on the other [68]. In the brain, apart from vascular cells, AhR is extensively expressed in neurons, astrocytes, and microglia [55,58]. The expression of AhR depends on the stage of development and is region- and cell-dependent [71].

The gut–brain axis, which involves interactions of intestinal ligands with peripheral and central AhR receptors, is considered an important element of proper brain function [63,68]. Microbiota-produced indoles, as well as many kynurenines, are recognized as potent AhR ligands [72]. However, despite several publications referring to IPA as an AhR ligand, the available data are ambiguous.

Studies that support IPA as an AhR agonist employed various experimental approaches. First, the effects of IPA were reversed in vivo by an AhR antagonist, as demonstrated, e.g., in a murine sepsis model [73]. Secondly, the level of AhR protein following exposure to certain indoles was increased, as shown in beta-amyloid (Aβ) precursor protein (APP)/PS1 mice in which a mixture of indoles, including IPA, upregulated the brain expression of AhR protein [43]. However, this effect was not specific to IPA, and the authors did not assess the affinity of the studied indoles to AhR. More precise conclusions were drawn in the study on an osteoarthritis model, where AhR knockdown abolished the IPA-evoked anti-inflammatory effects [74]. Other authors were unable to confirm the activation of AhR by IPA, as shown in 293T cells [75].

The net effect of IPA may depend on the presence of other ligands in the milieu. IPA blocked the effect of another AhR agonist, indoxyl sulfate, which is considered a uremic toxin. In cultured proximal tubular cells, IPA diminished the augmented expression of AHR, cytochrome P450, family 1, subfamily A, polypeptide 1 (CYP1A1), transforming growth factor β1 (TGF-β1), and monocyte chemoattractant protein-1 (MCP-1), possibly through the signal transducer and activator of transcription 3 (STAT3) suppression [76]. In at least two other studies, in an environment with different indoles present, the action of IPA on AhR was suggested to be unpredictable and varying [77,78]. Thus, considering the data gathered so far, the AhR receptor mediates the action of IPA. However, the degree of the effect is not fully predictable, since AHR signaling depends on other transcription factors, the proportion of various ligands in the extracellular milieu, and the cell type expressing the receptor.

The impact of IPA on the brain seems to be mediated by at least two pathways. First, intestinal IPA may alleviate local inflammation, and reduce the release of proinflammatory molecules, thus breaking the vicious cycle culminating in brain disorders. A rapidly growing amount of data supports the concept of the gut–brain axis viewed as the interaction of intestinal microbiota- and host cells-produced metabolites with the central nervous system. Special emphasis concerns the detrimental role of even subtle, yet chronic, inflammation. In such a scenario, a deficit of anti-inflammatory compounds may initiate processes culminating in morphological alteration and dysfunction of the brain. Secondly, when circulating IPA reaches the central compartment, it can directly modulate cellular processes through genomic, epigenetic, and receptor-linked switches.

### 3.2. Pregnane X Receptor

The pregnane X receptor (PXR) is encoded by the *nuclear receptor subfamily 1 group I member 2* (*NR1I2*) gene on chromosome 3q13.33 [79]. PXR is expressed within the cells of the enterohepatic system, immunocompetent cells such as lymphocytes, monocytes, or macrophages [80]. However, other cells and organs, including neurons and blood–brain-barrier endothelium, express PXR [81]. The primary action of PXR, similarly to AhR, relates to modulation of xenobiotics and drug metabolism and transport, e.g., through changes in the hepatic cytochrome P450, family 3, subfamily A (CYP3A) enzyme or the organic anion-transporting polypeptide 1a4 (Oatp1a4). Subsequently, drugs and xenobiotics are cleared from the body [82,83,84]. Again, similarly to AhR, PXR may exert non-genomic effects, including, e.g., interactions with NFkB, Toll-like receptors (TLRs), or the inflammasome. It also impacts cellular energetics and apoptotic processes [85,86].

An important aspect of brain PXR activation is associated with the metabolism of neurosteroids, which, in turn, may affect behavior and neuronal survival [87]. It is assumed that the neurotoxic effect of various xenobiotics may be in part mediated by PXR, which changes the expression of mitochondrial CYP450 and alters mitochondrial function [81]. Interestingly, there are significant inter-species differences in ligand affinity or selectivity in the case of PXR, similar to those in the case of AhR [86]. Moreover, the effects of PXR activation vary between species and cell types, and depend on the used ligand [81].

IPA seems to be a rather weak agonist for human PXR, with a half maximal effective concentration (EC_50_) of 120 μM. However, when co-administered with indole, IPA activates PXR in a much stronger manner. Mouse PXR is stimulated by IPA with much higher specificity (EC_50_ of 0.55 μM) [75]. In mouse models of PD, administration of IPA decreased the permeability of the intestinal barrier and blood–brain barrier (BBB), ameliorated inflammation, and improved dopaminergic neuronal function in a PXR-dependent manner, with a possible involvement of the Janus kinase 1 (JAK1)/STAT6 pathway [37].

### 3.3. Free Radical Scavenging

IPA is a hydroxyl radical scavenger acting over two-fold more potent than melatonin, a structurally and biochemically similar Trp derivative. The compound acts as an endogenous electron donor, scavenging free radicals effectively and preventing oxidative damage without undergoing autoxidation during the redox recycling of transition metals. It detoxifies highly reactive free radicals by donating electrons to hydroxyl anions [88,89,90].

IPA penetrates mitochondria, binds to complex I of the respiratory chain, and stabilizes energy metabolism, thereby lowering reactive oxygen species production [2]. Additionally, IPA has been observed to work in synergy with glutathione, another antioxidant, to inhibit the formation of cationic free radicals, further enhancing its protective effects [28]. IPA and its derivative, indolepropionamide (IPAM), reduced hydroxyl radical generation and inhibited the age-dependent impairment of mitochondrial function [91]. Furthermore, IPA protects DNA from oxidative injury, as shown in various models of DNA damage [92,93]. The action of IPA results from the prevention of lipid peroxidation and inhibition of the synthesis of proinflammatory cytokines [35,94,95]. Antioxidant effects of IPA are also evident in vivo. In Mongolian gerbils subjected to transient forebrain ischemia, orally administered IPA decreased lipid peroxidation in the hippocampus and reduced DNA damage in pyramidal neurons of the CA1 area [30].

### 3.4. Anti-Inflammatory Properties

Compelling data indicate that IPA acts as a potent anti-inflammatory molecule in various experimental paradigms [9,96]. The beneficial effects of IPA were demonstrated in liver steatosis, metabolic disorders, insulin resistance, cardiovascular diseases, and bone diseases [97,98,99].

The major mechanisms underlying the anti-inflammatory IPA effect comprise upregulated expression of AhR, decreased synthesis of the proinflammatory NF-κB, tumor necrosis factor-α (TNF-α), and other cytokines, reduced generation of the NLR family pyrin domain containing 3 (NLRP3) inflammasome, or enhanced synthesis of neurotrophic factors [41,96]. IPA may also inhibit the AKT protein pathway, with subsequent higher expression of the anti-inflammatory interleukin 10 (IL-10) and a decline in inflammation [100]. Recently, the ability of IPA to interfere with matrix metalloproteinase 9 (MMP9) was suggested [101].

IPA suppressed IL-1β-induced inflammation, extracellular matrix (ECM) degradation, and NF-κB signaling activation in chondrocytes in an AhR-mediated way [74]. AhR-dependent reduction in bacterial burden, protection against organ damage, and decreased sepsis-related mortality following IPA administration were shown [73]. IPA regulated PPT1 expression to modulate the PI3K-AKT-mTOR pathway, thereby restoring autophagic activity in senescent macrophages and suppressing both inflammation and aging-related myocardial fibrosis. Additionally, IPA influenced the cGAS-STING signaling pathway to regulate PPT1 expression [102].

Anti-inflammatory and neuroprotective effects of IPA are evident in the central nervous system. In primary human astrocytes, IPA attenuated the lipopolysaccharide (LPS)-evoked release of inflammatory cytokines, such as monocyte chemoattractant protein-1 (MCP-1), IL-12, IL-13, and TNF-α, yet it did not change the level of KYN or the KYN/Trp ratio [41]. A mixture of indole, indole-3-acetic acid, and IPA upregulated the expression of AhR, decreased the NF-κB and generation of the NLRP3 inflammasome, and lowered the level of proinflammatory cytokines in APP/PS1 mice, a mouse model of Alzheimer’s disease (AD). Indoles also improved the integrity of the intestinal barrier, enhanced cognition, and ameliorated brain neuropathology in APP/PS1 mice [43]. However, in the above study, the effect of IPA alone was not assessed.

Incubation of LPS-activated murine microglial BV2 cells in the presence of 1–5 μM IPA for 6 h diminished the release of proinflammatory TNF-α into incubation media. Furthermore, incubation with conditioned media from IPA-treated microglia restored BDNF and nerve growth factor (NGF) expression in neuroblastoma cells inhibited by LPS [33].

In mice, microdeletion of chromosomal region 16p11.2, known to contribute in humans to neurodevelopmental disorders, cognitive impairment, and dysbiosis, resulted in decreased IPA levels. In this model, orally administered IPA reversed hippocampal alterations and improved cognition, possibly as a result of an increased phosphorylation of ERK1 protein [53].

A study in 32 healthy elderly individuals receiving probiotics for 12 weeks revealed a 1.91-fold increase in serum IPA. IPA level was linked with bacterial diversity and with serum BDNF level. In cultured murine microglial cells treated with 5 μM IPA, the production of TNF-α decreased. Moreover, in human neuroblastoma SH-SY5Y cells, application of media from IPA-treated microglia enhanced BDNF and nerve growth factor synthesis [33].

### 3.5. KYNA

More than 95% of dietary Trp is degraded along the kynurenine pathway (KP) into derivatives collectively called kynurenines [103]. The metabolism of Trp starts with the conversion of N-formylkynurenine by two step-limiting enzymes, indoleamine 2,3-dioxygenase (IDO), and Trp 2,3-dioxygenase (TDO) [104]. The expression of IDO is induced by inflammatory mediators, e.g., interferon gamma (IFN-γ), IL-6, or TNF-α [105]. A novel way of the Trp metabolism through an enzyme, IL-4-induced 1 (IL4I1), acting as an L-amino acid oxidase, was recently described [106].

N-formylkynurenine is rapidly converted by formidase to the stable metabolite kynurenine, constituting the core of the pathway. Kynurenine is further metabolized along three major pathways. Firstly, it is transformed into KYNA by kynurenine aminotransferases (KATs) I-IV. Kynurenine can also be cleaved by kynureninase into anthranilic acid. Finally, kynurenine 3-monooxygenase (KMO) converts kynurenine to 3-hydroxykynurenine (3-HK). 3-HK is converted to xanthurenic acid or, through enzymatic and non-enzymatic conversion, with 3-hydroxyanthranilic acid as an intermediate, yields quinolinic acid (QUIN). The latter one is metabolized to nicotinamide adenine dinucleotide (NAD). Alternatively, 3-hydroxyanthranilic acid is converted to picolinic acid [103,107,108].

Kynurenines manifest a plethora of biological effects in peripheral and central compartments, including modulation of immune response, cellular survival, and metabolism (for a detailed review see: [109,110,111]). A broad array of data from various experimental paradigms revealed compromised function of the KP in cardiovascular, gastrointestinal, kidney tract, endocrine, or neuropsychiatric disorders, to name a few [64,112,113]. These discoveries resulted in various pharmacological attempts aimed at manipulating the KP, which is considered a promising therapeutic target in human pathology. However, the interplay between various kynurenines, and their net impact on a given cell, and the organ dynamically changes. It depends primarily on the efficacy of biosynthetic and catabolic processes and the proportion between metabolites known to exert the opposing action [114]. Interestingly, the oxidative status of the milieu seems increasingly important as it may alter the properties of metabolites from antioxidant to promoting oxidative stress [109].

The classical neurotoxic metabolites of the KP include QUIN, an endogenous agonist of glutamatergic receptors of N-methyl-D-aspartate type (NMDA), and 3-HK, which generates free radicals. However, anthranilic acid, 3-hydroxyanthranilic acid, xanthurenic acid, picolinic acid, or kynurenine itself may all generate free radicals [109,115].

On the other hand, KYNA is a pleiotropic molecule, for years considered an endogenous antagonist of glutamate receptors with neuroprotective properties [116]. With the years, the complex biological effects of KYNA have become unveiled [105,117]. Apart from the antagonism of iono- and metabotropic glutamate receptors, KYNA was reported to stimulate G-protein-coupled receptor 35 (GPR35), to act as a ligand of adrenoceptor alpha 2B (ADRA2B) and hydroxycarboxylic acid receptor 3 (HCAR3), and to stimulate the α-7 nicotinic acetylcholine receptor (α7nAChR), although the latter effect is a matter of debate [64,105,110].

Furthermore, KYNA, similarly to other metabolites of the KP, such as kynurenine or xanthurenic acid, is an agonist of AhR, yet with higher potency than other kynurenines, showing an ED_50_ of 100 nM-1 μM [105,118,119].

KYNA occurs in the serum, CSF, and various organs, including the brain. The concentration is within the 40–100 nM range. However, KYNA may attain much higher levels locally, for example, during inflammation [105]. The classical KYNA synthesis pathway in the brain involves the conversion of L-kynurenine by KATs, and was initially ascribed to astroglial cells, but it is now believed that microglial and neuronal cells may also synthesize KYNA [120]. It is noteworthy that peripheral KYNA practically does not reach the central compartment [121].

Therefore, attempts to alter central KYNA level were aimed mostly to increase the brain concentration of KYNA substrate, kynurenine, or to use compounds that, upon penetration through the blood–brain barrier, may change the activity of KATs, or impede the conversion of kynurenine along the other two arms of the KP [107,122].

Recently, it was demonstrated that orally administered IPA may boost serum and brain KYNA, but not kynurenine levels in rodents [34]. Moreover, sub-chronic treatment with IPA provided in the chow caused several-fold increases in both IPA and KYNA levels in the plasma and prefrontal cortex of the animals. In contrast, KYNA levels in medial prefrontal cortex and striatum were not affected by in situ IPA administration via microdialysis probe. Similarly, the addition of IPA to homogenates of rat prefrontal cortex tissue and recombinant human KATII did not influence KATII activity in concentrations up to 1 mM [34]. Thus, IPA is a potent stimulator of KYNA production, acting most probably within the intestinal compartment. However, the precise mechanisms underlying this effect remain to be elucidated.

## 4. IPA and Neurodegeneration

Acute or chronically progressing neuronal loss is a hallmark of brain disorders such as stroke, trauma, neurodegenerative, or autoimmune diseases [123,124]. Underlying pathogenesis includes a network of processes triggered by exogenous, environmental, and genetically determined factors. An excessive release of glutamate enhanced intracellular Ca^2+^ accumulation, and disturbed mitochondrial function, together with free radical generation, and abnormal buildup of misfolded proteins culminate in neuronal cell death [3,125,126,127]. Despite tremendous scientific efforts, currently approved drugs impact neurodegeneration in a limited way. The treatment is restricted to partial mitigation of the neuronal loss during acute brain events. In chronic neurodegeneration, therapies are symptomatic, able to ameliorate the manifestations of some disorders, but not to stop or reverse the course of the disease. There is an urgent need for the development of novel prophylactic and disease-modifying treatments.

The increasing understanding of the role played by intestinal microbiota and their metabolites in the function of the brain has resulted in the gut–brain hypothesis of neurodegeneration [128,129]. Accordingly, a number of interventions were proposed to improve the diversity of microbiota, to boost the population of beneficial species, and to use selected microbial metabolites as potential drugs [129].

In view of the available data, IPA has emerged as a therapeutic molecule with promising neuroprotective potential. The compound, either produced by the microbiota or delivered orally, can easily cross the BBB and impact neuronal function through several processes described above, with subsequent changes in neurotransmission, impairment of inflammation, and protection against free radical-induced damage [35,94,95]. Peripherally administered IPA easily reaches the brain and may attain high levels [14,28,29,30].

Protection against neurodegeneration offered by IPA, particularly during high oxidative stress, inflammation, and in the processes linked to the abnormal aggregation of proteins, was demonstrated in various experimental paradigms [2,10,42,130].

In HT22 cells, an immortalized murine hippocampal cell line, IPA reduced apoptosis and oxidative damage caused by lipopolysaccharide (LPS) [131]. It also decreased neuroinflammation by inhibiting the receptor for advanced glycation end-product (RAGE)/JAK2/STAT3 signaling pathway in LPS-treated BV2 cells, a murine microglial cell line [131]. This pathway is associated with neuronal injury, inflammatory responses, and oxidative stress [132,133]. IPA derivatives demonstrated neuroprotective and antioxidant activities and inhibited MAO-B in SH-SY5Y neuroblastoma cells [36].

### 4.1. Alzheimer’s Disease (AD)

AD, the major cause of dementia in the elderly, is predicted to affect 150 million people worldwide by the year 2050 [134]. It is characterized by the progressive accumulation of Aβ in amyloid plaques in the brain and vasculature, and by deposits of hyperphosphorylated tau protein within neurons, neurofibrillary tangles, and increasing loss of neurons and cognitive abilities [135,136]. The social and economic costs of disease are tremendous, and, despite our increasing understanding of the underlying mechanism, currently available therapeutic options are merely symptomatic. Novel drugs introduced to therapy and targeting amyloid plaques, such as lecanemab and donanemab, offer moderate improvement in the brain pathology and cognition at the early stage of the disease; furthermore, they are linked with several serious adverse effects [137].

The role of gut dysbiosis in the development of AD was the subject of numerous studies, presented in recent reviews [2,10,138]. Experimental data support the view of IPA as beneficial in AD. The initial discovery came from studies in neuroblastoma cells and in neuronal primary cultures, in which IPA prevented oxidative stress and inhibited Aβ-induced cell death [35]. In neuroblastoma cells expressing the human APP gene, IPA increased mitochondrial membrane potential and respiration rate, and reduced free radical production [138]. Recently, in silico and in vitro research demonstrated that esters of IPA inhibit acetylcholinesterase (AChE) and butyrylcholinesterase (BChE) activities, which are pharmacological targets in AD [139,140].

IPA reduced Aβ fibril formation by 50% and reduced the stimulatory effect of apolipoprotein E4 (ApoE4), measured by fluorescence intensity (unpublished observation of Pappolla et al. cited in a review by [141]). Melatonin, a compound structurally similar to IPA, exerts corresponding effects, as it reverses the generation of neurofibrillary tangles evoked by ApoE4 on Aβ [142]. Both IPA and melatonin were demonstrated to improve mitochondrial respiratory rate in APP-expressing neuroblastoma cells [138]. Melatonin has antioxidant properties, augments Aβ clearance by the lymphatic system, and decreases oligomeric Aβ40 in the brain [3,143,144]. The effect of both IPA and melatonin is cumulative when it comes to preventing lipid peroxidation [145].

In vivo, a strong positive correlation was observed between the levels of IPA generated by gut microbiota and behavioral changes in the 5xFAD transgenic AD mouse model [44]. A 30-day treatment with *Clostridium sporogenes* improved cognitive performance and spatial memory, restored the synaptic ultrastructure and attenuated neuroinflammatory responses, and reduced cortical and hippocampal Aβ accumulation. Concomitant increase in IPA levels, the relative prevalence of IPA-synthesizing bacteria in the gut, and a decrease in bacteria characteristic for AD, e.g., *Aquabacterium*, *Corynebacterium*, and *Romboutsia,* were shown [44]. In transgenic APP/PS1 mice fed a Trp-free diet and treated orally with indole, IAA, and IPA for 4 weeks, a prominent reduction in inflammatory markers was observed. Upregulated AhR, inhibition of the NF-κB pathway, and decreased formation of the NLR family pyrin domain containing 3 (NLRP3) inflammasome, reduced the levels of TNF-α, IL-6, IL-1β, and IL-18, and alleviated the inflammatory response, were all observed [43]. IPA also ameliorated cognitive deficits and neuroinflammation in LPS-treated mice, possibly by inhibition of the RAGE/JAK2/STAT3 signaling pathway [131]. In the murine APP/PS1 AD model, IPA was shown to interact with peroxisome proliferator-activated receptor α (PPARα) and to activate neuroprotective genes, i.e., *Bdnf*, *Ppar*α, *acyl-CoA synthetase, bubblegum family, member 1 (Acsbg1)*, *stearoyl-CoA desaturase 2 (Scd2)*, and *Scd3* [45]. In male mice, increased abundance of indole-producing bacteria *Lactobacillus reuteri,* elevated levels of IPA, and enhanced PPARα signaling were linked an improvement of working memory and reduction of neuronal damage [45]. Tetradecyl 2,3-dihydroxybenzoate (ABG-001), a molecule with NGF-mimicking activity and anti-aging properties, enhanced the production of IPA by gut microbiota in a murine model of AD. In this study, IPA was shown to target the heat shock cognate 70 kDa protein (Hsc70) and regulate the signaling pathways Hsc70/pyruvate kinase muscle isozyme/hexokinase 2/light chain 3 (PKM2/HK2/LC3) and forkhead box O3/sirtuin 1 (FOXO3a/SIRT1). ABG-001 diminished the memory dysfunctions of the experimental animals by modulating inflammation through IPA and autophagy via Hsc70 [46]. Intermittent fasting, increasingly recognized as cardioprotective, neuroprotective, and promoting longer life span, was shown to improve mitochondrial bioenergetics, and to reverse cognitive deficits in diabetic mice. Alterations in microbial metabolites included an increase in IPA levels [47]. Furthermore, the effects of intermittent fasting were mimicked by intraperitoneal administration of IPA [47].

To our knowledge, just one study reported contrasting results. In patients with mild cognitive impairment, metabolomics and in silico studies suggested that the IPA level is a predictor of progression to AD. However, the trend towards increased plasma IPA was statistically insignificant, and the conclusion was based on the model constructed with the use of eight metabolites [58].

In 2002, IPA was suggested as a promising therapeutic agent in AD [141]. Plans for pre-clinical and clinical studies were also mentioned [141]; however, no data about such clinical trials are available. In a recent review, the authors proposed that the health of the aging brain can be promoted, and dementia development may be prevented by inducing a higher production of indoles, such as IPA, by the microbiota [2].

### 4.2. Parkinson’s Disease (PD)

PD is the second most prevalent neurodegenerative disorder [146]. The accumulation and toxic aggregates of α-synuclein and the degeneration of dopaminergic neurons in the substantia nigra are hallmarks of the disease [147,148,149]. Genetic and environmental factors, which result in oxidative stress, decreased antioxidant capacity, neuroinflammation, lipid peroxidation, mitochondrial dysfunction, and pathological aggregation of α-synuclein, have been implicated in PD pathogenesis [149,150,151,152].

Therapeutic options are symptomatic and involve enhancement of central dopaminergic transmission and acetylcholinesterase inhibitors (AChE-I) [153]. Increasing evidence implies gut microbiota as a potential therapeutic target in PD [64,154].

IPA has been demonstrated to act as a chemical chaperone, reducing endoplasmic reticulum (ER) stress in cells that overexpress Parkin-associated endothelin-like receptor (GPR37) and α-synuclein, thus modeling PD [42]. IPA derivatives demonstrated neuroprotective and antioxidant activities in neuroblastoma cells and inhibited MAO-B, an enzyme that oxidizes dopamine, thus depleting its level and generating free radicals [36]. Furthermore, IPA esters were shown to inhibit the AChE and BChE activities [139]. In rotenone-stimulated enteric glial cells, IPA, in a PXR-mediated manner, reduced mRNA expression of IL-6, IL-1*β*, and TNF-*α*, impaired IL13 signaling with subsequent decline of JAK1-STAT6 pathway [37]. The protective effects of IPA were also evident in vivo, in a rotenone model of PD in mice, where IPA alleviated enteric gliosis and restored the integrity of the intestinal and brain barriers [37].

A study on 56 PD patients revealed surprisingly higher IPA levels in the plasma compared to controls (1.26 vs. 0.83 μM); however, IPA did not correlate with cognitive and motor status scores of the patients [27]. Furthermore, the analyses were not performed separately for patients with early (N = 19) and advanced (N = 37) disease, and the impact of medications and diet was not taken into consideration [27]. The elevation of plasma IPA in PD patients could be a compensatory protective mechanism.

### 4.3. Hypoxia/Ischemia

IPA may also protect the brain after hypoxia and ischemia, as has been shown in several studies. IPA had a protective effect on the disruption of the blood–brain barrier following hypoxic-ischemic brain injury in neonatal rats, possibly by modulating the PXR signaling pathway [39]. Indeed, its protective effects were reversed by PXR antagonists [39]. In this study, IPA alleviated inflammation by inhibiting the NF-κB pathway. This, in turn, reduced the brain injury-evoked expression of inducible nitric oxide synthase (iNOS), TNF-α, IL-6, and IL-1β, and decreased intracellular ROS levels. PXR overexpression in the rat brain microvascular endothelial cells ameliorated the decline in junctional proteins expression. Furthermore, it reduced the activity of the NF-κB pathway in the model of oxygen-glucose deprivation injury. Additionally, IPA downregulated the activation of MMPs in rat brain microvascular endothelial cells [39].

In a study on Mongolian gerbils, 2-week oral pretreatment with IPA reduced hippocampal CA1 damage by 50%, decreased expression of proinflammatory markers, and ameliorated DNA damage in pyramidal cells [30]. In a murine model of middle cerebral artery occlusion (MCAO), post-stroke intragastric IPA ameliorated neuroinflammation and neuronal damage [48]. It also improved the composition of gut microbiome with subsequent recovery of intestinal permeability and modulated the activity of regulatory T (Treg) and T helper 17 (Th17) cells in intestinal lymphoid tissue [48]. Boosting microbial IPA production by acupuncture exerted beneficial effects in a glucose/oxygen deprivation model [155].

A clinical study on 197 patients with acute cerebral infarction revealed that low serum IPA is an independent predictor of acute stroke and poor prognosis [59]. In line with this report, it was recently discovered that mice bacterial phyla, known to generate AHR ligands, are significantly reduced by aging and experimental stroke. Furthermore, in the middle cerebral artery occlusion stroke model, a decreased serum IPA in mice was observed [49]. Conversely, IPA treatment reduced the expression of IL-1β, IL−4, and IL-10 in microglial cells exposed to oxygen-glucose deprivation. In the same study it was shown that, in patients suffering from stroke, plasma IPA was decreased up to 7 days after the event [49].

### 4.4. Other Disorders

To our knowledge, there is only a single report on IPA status in patients with HD. The study included patients with premanifest (N = 52) and early symptomatic (N = 102) HD. Plasma IPA levels were decreased in both groups, reaching 138.5 ng/mL and 107.7 ng/mL, respectively, vs. control (191.1 ng/mL; N = 140) [25].

The data on the role of IPA in multiple sclerosis are ambiguous. An increased IPA synthesis was observed in the relapsing–remitting experimental autoimmune encephalomyelitis in mice, considered a multiple sclerosis model [156]. Similarly, higher IPA was observed by others in patients with the relapsing–remitting course of multiple sclerosis [60]. Furthermore, Expanded Disability Status Scale (EDSS) scores, demonstrating the disability level in patients with multiple sclerosis, were significantly correlated with the urine concentration of IPA (r = 0.5, *p* < 0.001) [60]. On the other hand, activation of astrocytic AHR by IPA was shown to reduce the central nervous system inflammation and to decrease immune-mediated damage in experimental autoimmune encephalomyelitis [54].

IPA’s protective properties were observed in models of autism spectrum disorder. IPA deficiency disrupts the IPA/AHR/NF-κB signaling pathway, resulting in the overactivation of hippocampal microglia and excessive pruning of neuronal synapses. This dysregulation is associated with neural patterns and behaviors similar to those observed in autism spectrum disorder [52].

IPA was also found to promote axon regeneration and functional recovery after sciatic nerve crush in mice [56]. Administration of IPA for 10 days before injury and up to the third day after injury enhanced axonal regeneration and the recovery of sensory function. IPA increased the expression of the PXR gene and genes involved in neutrophil chemotaxis, thereby regulating the regenerative capacity of dorsal root ganglia. Moreover, the inhibition of neutrophil chemotaxis prevented IPA-mediated axonal regeneration [56].

In a rat traumatic brain injury model, the serum levels of IPA exhibited an initial downward trend, which became reversed after three days [57]. These results suggested that IPA may serve to monitor the time that elapsed from the injury [57].

Research on the potential beneficial effects of IPA in aging revealed that elevated IPA levels were associated with an increase in serum BDNF in healthy elderly subjects [33].

## 5. Conclusions

It is generally well established that administration of IPA to experimental animals or humans is not associated with significant adverse effects. In contrast to other antioxidants, the advantage of IPA is that it does not exhibit pro-oxidant activity, as it does not undergo decarboxylation of the side chain to produce reactive peroxy-radicals [157]. IPA generated as part of normal metabolism, at physiological levels, primarily exerts no cytotoxic effect [10,138,158]. The lack of toxicity in neuroblastoma cells was shown up to 500 μM [36]. Furthermore, IPA derivative was shown to extend life span in experimental animals and exert protective effects in various models, ranging from cell loss due to cytostatic use through myocardial fibrosis up to brain neurodegeneration [10,91]. It seems of interest, though, that IPA in high doses may inhibit the growth of some neoplastic cells [159].

However, IPA acts as a plant auxin and can be enriched in a variety of vegetables and fruits, with the consequences for humans not fully understood [160]. AhR is crucial in prenatal development, but, on the other hand, it was implicated in age-related pathologies [161]. Furthermore, AhR may control cellular fate by acting as a neurogenesis-promoting factor. However, it may also be linked with morphological and functional brain changes during aging [162,163].

Prolonged excessive AhR stimulation was related to oncogenic potential. Gastric glandular epithelial tumors in mice with constitutively active AhR were reported [164]. In contrast, IPA itself does not seem to contribute to tumor development. In fact, the cytostatic properties of IPA, related to the activation of AhR and PXR, were demonstrated, further supporting the beneficial effects of IPA administration [165]. Thus, the contribution of AhR to IPA action requires further studies, especially in the context of potential adverse effects of long-term therapy.

According to the ClinicalTrial.gov database, three studies aimed to evaluate the effects of IPA in humans. Single or chronic oral administration of IPA (150 mg) was examined in patients with Friedreich’s ataxia, and no toxic effects were noted [166]. Another study on the toxicity of IPA, Indole-3-PROpionic Acid Clinical Trials-a Pilot Study (iPROACT-pilot), was recently finished, but no results have been posted yet [167]. The third trial, Tryptophan for Impaired AhR Signaling in Celiac Disease (TIARSCeD), is currently ongoing [168].

Microbiota-generated IPA displays pleiotropic effects associated with its ability to target AhR and PXR, to ameliorate the inflammation, and reduce free radical toxicity. The beneficial outcome of experimental IPA administration in AD, PD, and stroke models is well substantiated. Nutritional factors, specifically a low-fat, high-fiber, high-carbohydrate, and Trp-rich diet, along with probiotic supplementation, may enhance gut IPA synthesis. Thus, a well-planned diet may be beneficial in the course of neurodegeneration, considering its effect on IPA and the gut–brain axis.

### Future Directions

There are still a number of unanswered questions concerning IPA action. The majority of data implicate the beneficial effects of IPA, yet some findings warrant further research. It is not clear whether IPA will offer clinical improvement in multiple sclerosis. Furthermore, the contribution of AhR activity to neuronal survival should be studied in more detail, including potentially confounding factors such as diet composition, microbiome variability, and pharmacokinetics of the compound. Similarly, the impact of IPA on KYNA synthesis must be clarified. Currently, it is not understood what intestinal factors are necessary to stimulate the generation of KYNA, and whether they are of microbial or intestinal origin.

In view of the accumulated data and considering a huge demand for novel neuroprotective compounds, clinical trials evaluating the potential use of IPA are essential. We suggest that IPA should be evaluated as (1) a preventive measure aimed to delay the onset of AD- and PD-related neurodegeneration and cognitive impairment in high-risk populations, (2) a therapeutic add-on intervention aimed to slow down the progress of AD and PD, and (3) a therapeutic approach aimed to limit the morphological and behavioral consequences of ischemic stroke. Further experimental and clinical research should also bring more details on the prospective use of IPA in other clinical conditions associated with neuronal loss.

## Figures and Tables

**Table 1:** Neuroprotective effects of IPA in preclinical studies.

Subjects	Model	Experimental Paradigm	Outcome	Ref.
**In vitro studies**				
Primary hippocampal neurons; neuroblastoma cells	AD model-exposure to Aβ.	1 µM IPA for 24 h.	IPA inhibited Aβ-induced lipid peroxidation, prevented neuronal death.	[35]
Neuroblastoma cells; mouse brain endothelial cells	Neurodegeneration model-induced.	IPA-derived and 5MICA-derived hydrazone hybrids tested at up to 200 μM.	IPA derivatives protected neurons against oxidative stress, inhibited MAO-B, and preserved BBB integrity—supporting their neuroprotective potential.	[36]
Enteric glial cells	PD model-rotenone (600 nM, 72 h).	IPA at 0.1 μM co-administered with rotenone.	IPA inhibited IL13Rα1/JAK1/STAT6 signaling.	[37]
BV2 microglial cells and SH-SY5Y neuronal cells	LPS-induced inflammation.	5 μM IPA for 6 h.	IPA reduced release of TNF-α.	[33]
Hippocampal HT-22 neurons	H_2_O_2_-induced oxidative stress.	pretreatment with or without GPR30 antagonist of G15 for 2 h, incubation with IPA (250 and 500 µm) for 24 h.	IPA alleviated neurodegeneration upregulation of AMPK/SIRT1 pathway, and neuronal apoptosis.	[38]
Rat brain microvascular endothelial cells	BBB model.	pretreatment with 2 mM IPA in DMSO for 2 h before OGD.	IPA reduced oxidative stress, MMP activity, and apoptosis; effect mediated via activation of PXR and inhibition of NF-κB signaling.	[39]
hCMEC/D3 endothelial cells	BBB model.	IPA 1 µM for 24 h + Ox-LDL 50 µg/mL for 12 h.	IPA preserved BBB integrity, improved endothelial function, and exerted protective effects via FFAR3 activation.	[21]
Astrocytes	Release of gliotransmitters which impact cell viability.	IPA 0.001–0.1 mM for 10 min; pre-treatment 24 h.	No effect on ATP and glutamate release from astrocytic cells.	[40]
Astrocytes	LPS-induced cytotoxicity.	IPA (50 μM) or butyrate before LPS (1 μg/mL) stimulation.	IPA decreased LPS-evoked increases in MCP-1, IL-12, IL-13, and TNF-α levels.	[41]
Neuroblastoma cells	PD model-Pael-R and α-synuclein overexpression in tunicamycin-induced apoptosis model.	0.1–1 mM IPA treatment.	IPA suppressed protein aggregation, reduced ER stress, and protected neurons from apoptosis.	[42]
**In vivo studies**				
APP/PS1 transgenic mice	AD model.	Oral gavage; indole + IAA + IPA at 20 mg/kg/day for 4 weeks.	IPA (with other indoles) activated AhR, inhibited NF-κB/NLRP3 signaling, reduced release of proinflammatory cytokines and neurodegeneration.	[43]
5xFAD transgenic mice	AD model.	Clostridium sporogenes (1 × 10^10^ CFU/day) + xylan (1% *w*/*w*) orally, for 30 days.	Improved cognition and memory, reduced Aβ pathology, enhanced synaptic structure, increased IPA levels and IPA-producing bacteria; reduced neuroinflammation, restored gut barrier integrity.	[44]
APP/PS1 male mice	AD model.	16-week methionine-restricted diet (0.17% *w*/*w*).	Increased serum IPA, improved cognition, reduced neuronal damage. IPA activated PPARα signaling and enhanced gut barrier integrity.	[45]
mice	AD model-high-fat diet induced.	ABG-001 administered orally at 50 mg/kg/day for 30 days.	ABG-001 increased IPA, IPA targeted heat shock cognate 70 pathway (Hsc70/PKM2/HK2/LC3 and FOXO3a/SIRT1); reduced neuroinflammation.	[46]
C57BL/6J mice	Diabetes-induced cognitive impairment.	IPA administered orally (10 mg/kg/day for 28 days).	IPA improved cognitive function, and reduced neuroinflammation.	[47]
Mice	Age-related neurodegeneration model (d-galactose).	0.5% CMC-Na dissolved in 0.9% NaCl in oral gavage, galactose s.c. 300 mg/kg/day for 8 weeks, 50 mg kg^−1^ IPA.	IPA alleviated neurodegeneration by reducing oxidative stress, inflammation, and neuronal apoptosis.	[38]
C57BL/6J mice	PD model-rotenone (30 mg/kg, i.g., 4 weeks).	IPA orally at 25, 50, or 100 mg/kg/day for 6 weeks.	IPA reduced EGC gliosis, neuroinflammation, and intestinal/brain barrier damage.	[37]
C57BL/6 mice	MCAO model of ischemic stroke.	IPA intragastrically administration at 400 μg/20 g/day during MCAO.	IPA restored gut microbiota composition, enhanced intestinal barrier integrity, modulated Treg/Th17 balance, reduced neuroinflammation and infarct size, and improved neurological function.	[48]
Wild-type and germ-free mice	MCAO model of ischemic stroke.	IPA (50 mg/kg/day, oral gavage) given for 14 days post-stroke.	IPA treatment reduced neuroinflammation, improved neurological function, and rebalanced AHR ligand pools derived from host (kynurenine pathway) and microbiota (tryptophan catabolites).	[49]
Aged C57BL/6 mice	Postoperative delirium-like behavior induced by anesthesia/surgery.	IPA (0.0625 mmol/kg) in 0.9% NaCl:ethanol (*v*/*v* 10:1) injected 2×/day at	IPA modulated aberrant hippocampal neural activity and reduced delirium-like behavior in aged mice.	[50]
C57BL/6 mice	Sensorineural hearing loss induced by TCP exposure.	40 mg/kg of IPA orally for 21 days.	IPA preserved hearing and cochlear structure, reduced oxidative stress, and activated immune defense via neutrophil and IFN-γ signaling.	[51]
Rats	Intrauterine growth retardation model of autism spectrum disorder.	IPA orally administered at 20 mg/kg/day for 4 weeks starting at postnatal week 4.	IPA ameliorated autism-related behaviors, normalized microglial synaptic pruning, reversed NFκB upregulation, and upregulated synaptic markers PSD95 and SYN.	[52]
Mice with 16p11.2 deletion	Autism spectrum disorder model.	Oral IPA (20 mg/kg/day) or vehicle from postnatal day 42 for 2 weeks, followed by behavioral testing.	16p11.2 mice exhibited altered gut microbiota and reduced IPA levels; IPA supplementation improved memory and social behavior and restored inhibitory signaling in the hippocampus.	[53]
C57BL/6 mice	BBB function.	IPA 100 mg/kg/day orally for 12 weeks (in vivo); dietary Trp supplementation (0.1%/0.5%).	IPA preserved BBB integrity, improved endothelial function, and exerted protective effects via FFAR3 activation.	[21]
Neonatal Sprague-Dawley rats	rBMEC cells	IPA (15 mg/kg, IP) 2×/day (2 days before ligation of the common carotid artery, and a after operation and hypoxia).	IPA preserved BBB integrity by reducing inflammation, oxidative stress, MMP activity, and apoptosis; effect mediated via activation of PXR and inhibition of NF-κB signaling.	[39]
C57BL/6J, Ifnar1^−/−^, IL-27ra^−/−^, GFAP-Cre, and AhR^fl/fl^ mice	Experimental EAE model of MS.	IPA administered via oral gavage at 400 μg/20 g body weight/day daily from day 22 after induction of EAE.	Administration of IPA alleviated EAE symptoms in AhR-dependent way.	[54]
Female rats	Neurotoxicity induced by epirubicin.	Co-administration of IPA at 20 or 40 mg/kg/day orally for 28 days.	IPA protected against epirubicin-induced neurotoxicity by modulating oxidative and inflammatory pathways.	[55]
Sprague–Dawley rats	Trp pathway.	Oral IPA at 200 mg/kg; subchronic feeding at 100 mg/day or 350 mg/day for 7 days.	IPA increased brain and plasma levels of IPA and KYNA; Subchronic feeding raised plasma IPA ~19–27× and brain IPA ~2–3×.	[34]
Male C57BL/6 mice	Sciatic crush model of peripheral nerve injury.	Oral gavage or i.p. injection of IPA; 10 or 20 mg kg^−1^ per day.	IPA production by *Clostridium sporogenes* was required for efficient axonal regeneration; IPA delivery after sciatic injury accelerated sensory function recovery.	[56]
Sprague–Dawley rats	Traumatic brain injury model.	Serum IPA level determination	IPA serum levels initially exhibited a downward trend; after 3 days, the trend was reversed.	[57]

Abbreviations: 5-MICA, 5-methoxy-1H-indole-3-carbonitrile; ABG-001, tetradecyl 2,3-dihydroxybenzoate; AD, Alzheimer’s disease; AhR, aryl hydrocarbon receptor; AMPK, 5′AMP-activated protein kinase; Aβ, β-amyloid; BV2, mouse microglial cell line; CFU, Colony Forming Unit; DMSO, dimethyl sulfoxide; EAE, autoimmune encephalomyelitis; EGC, enteric glial cells; FFAR3, free fatty acid receptor 3; FOXO3a, forkhead box O3; GPR30, G protein-coupled receptor 30/G protein-coupled estrogen receptor 1; GPX4, glutathione peroxidase 4; H_2_O_2_, hydrogen peroxide; CMEC/D3, immortalized human brain microvascular endothelial cell line; HK2, hexokinase 2; Hsc70, heat shock cognate 70 kDa protein; HT-22, mouse hippocampal neuronal cell line; IL-12/13, interleukin 12/13; IL13RA1, interleukin 13 receptor subunit alpha 1; IPA, indole-3-propionic acid; JAK1, Janus kinase 1; KYNA, kynurenic acid; LC3, light chain 3; LPS, lipopolysaccharide; MCAO, middle cerebral artery occlusion; MCP-1, monocyte chemoattractant protein-1; MI/R, myocardial ischemia-reperfusion; MMP, matrix metalloproteinase; MS, multiple sclerosis; NF-κB, nuclear factor kappa-light-chain-enhancer of activated B cells; NLRP3, NLR family pyrin domain containing 3; Nrf2, nuclear factor E2-related factor 2; OGD, oxygen and glucose deprivation; PD, Parkinson’s disease; PKM2, pyruvate kinase muscle isozyme; PPARα, peroxisome proliferator-activated receptor α; PS, presenilin 1; PSD-95, postsynaptic density protein 95; PXR, pregnane X receptor; rBMECs, rat brain microvascular endothelial cells; SH-SY5Y, human neuroblastoma cell line; SIRT1, sirtuin 1/silent information regulator 1; STAT6 signal transducer and activator of transcription 6; SYN, synuclein; TCP, 3,5,6-Trichloro-2-pyridinol; Th17, T helper 17 cells; TNF-α, tumor necrosis factor-α; Treg, regulatory T cells.

**Table 2:** Effects of IPA on neurodegeneration in clinical studies.

Patients	Controls	Outcome	Ref.
Mild cognitive impairment patients who proceeded to AD (*n* = 19).	Patients with stable mild cognitive impairment (*n* = 29).	An insignificant increasing trend of plasma IPA from stable mild cognitive impairment to AD.	[58]
Patients with PD (*n* = 56).	Age- and sex-matched healthy participants (*n* = 43).	Higher IPA levels in the plasma of PD patients compared to controls (1.26 vs. 0.83 μM); no correlation of IPA with cognitive and motor status scores of the patients.	[27]
Patients with stroke (*n* = 60).	Age-matched controls without stroke (*n* = 64).	Decreased serum IPA from 1 to 7 days after stroke.	[49]
Patients with acute cerebral infarction (*n* = 197).	Participants from a community-based stroke screening program (*n* = 53).	Low serum IPA served as an independent predictor of acute stroke and poor prognosis.	[59]
Patients with premanifest (*n* = 52) and early symptomatic (*n* = 102) HD.	Healthy controls (*n* = 140).	Decreased plasma IPA levels in both groups: 138.5 ng/mL (premanifest HD) and 107.7 ng/mL (early symptomatic HD) vs. control (191.1 ng/mL).	[25]
Patients with relapsing–remitting multiple sclerosis (*n* = 47).	Healthy controls (*n* = 43).	EDSS scores were significantly correlated with the urine concentration of IPA (r = 0.5, *p* < 0.001).	[60]
Healthy elderly individuals (≥65 years of age) receiving probiotics (*n* = 32).	Healthy elderly individuals (≥65 years of age) receiving placebo (*n* = 31).	Elevated IPA levels were positively associated with serum BDNF levels in the probiotics group (r = 0.28, *p* < 0.05).	[33]

Abbreviations: AD, Alzheimer’s disease; BDNF, brain-derived neurotropic factor; EDSS, Expanded Disability Status Scale; HD, Huntington’s disease; IPA, indole-3-propionic acid; PD, Parkinson’s disease.

## Data Availability

No new data were created or analyzed in this study. Data sharing is not applicable to this article.

## Abbreviations

The following abbreviations are used in this manuscript:

3-HK	3-hydroxykynurenine
ABG-001	tetradecyl 2,3-dihydroxybenzoate
acdA	acyl-coenzyme A dehydrogenase
AChE	acetylcholinesterase
ACSBG1	acyl-CoA synthetase, bubblegum family, member 1 gene
AD	Alzheimer’s disease
ADRA2B	adrenoceptor alpha 2B
AhR	aryl hydrocarbon receptor
Apo-E4	apolipoprotein E4
APP	amyloid-beta precursor protein
ArAT	aromatic amino acid aminotransferase
Aβ	β-amyloid
BBB	blood–brain barrier
BChE	butyrylcholinesterase
BDNF:	brain-derived neurotrophic factor
CSF	cerebrospinal fluid
CYP1A1	cytochrome P450, family 1, subfamily A, polypeptide 1
CYP3A	cytochrome P450, family 3, subfamily A
EC_50_	half maximal effective concentration
ECM	extracellular matrix
EDSS	Expanded Disability Status Scale
fldBC	phenyllactate dehydratase BC
FOXO3a	forkhead box O3
GPR	G-protein-coupled receptor
HCAR3	hydroxycarboxylic acid receptor 3
HD	Huntington’s disease
HK2	hexokinase 2
Hsc70	heat shock cognate 70 kDa protein
IA	indoleacrylic acid
IAA	indole-3-acetic acid
IDO1	indoleamine 2,3-dioxygenase 1
IFN-γ	interferon gamma
IL-1β/4/6/10	interleukin 1β/4/6/10
IL4I1	interleukin 4-induced 1
ILA	indolelactic acid
ILDH	indole-lactate dehydrogenase
iNOS	inducible nitric oxide synthase
IPA	indole-3-propionic acid
IPAM	indolepropionamide
iPROACT-pilot	Indole-3-PROpionic Acid Clinical Trials-a Pilot Study
IPyA	indole-3-pyruvic acid
JAK1/2	Janus kinase 1/2
KATs	kynurenine aminotransferases
KMO	kynurenine 3-monooxygenase
KP	kynurenine pathway
KYNA	kynurenic acid
LC3	light chain 3
LPS	lipopolysaccharide
MAO	monoamino-oxidase
MCAO	middle cerebral artery occlusion
MCP-1/CCL2	monocyte chemoattractant protein-1
MMP	matrix metalloproteinase;
NAD	nicotinamide adenine dinucleotide
NF-κB	nuclear factor kappa-light-chain-enhancer of activated B cells
NGF	nerve growth factor
NLRP3	NLR family pyrin domain containing 3
NMDA	N-methyl-D-aspartate type
NR1I2	nuclear receptor subfamily 1 group I member 2 gene
Oatp1a4	organic anion-transporting polypeptide 1a4
PD	Parkinson’s disease
PKM2	pyruvate kinase muscle isozyme
PPARα	peroxisome proliferator-activated receptor α
PXR	pregnane X receptor
QUIN	quinolinic acid
RAGE	receptor for advanced glycation end-products
ROS	reactive oxygen species
SCD2/3	stearoyl-CoA desaturase 2/3 gene
SIRT1	sirtuin 1
STAT3/6	signal transducer and activator of transcription 3/6
TAA	Trp aminotransferase
TDO	tryptophan 2,3-dioxygenase
TGF-1	transforming growth factor β1
Th17	T helper 17 cells
TIARSCeD	Tryptophan for Impaired AhR Signaling in Celiac Disease
TLRs	Toll-like receptors
TNF-α	tumor necrosis factor-α
Treg	regulatory T cells
Trp	tryptophan
α7nAChR	α-7 nicotinic acetylcholine receptor
